# Synthesis of Luminescent Carbon Dots with Ultrahigh Quantum Yield and Inherent Folate Receptor-Positive Cancer Cell Targetability

**DOI:** 10.1038/s41598-018-19373-3

**Published:** 2018-01-18

**Authors:** Haifang Liu, Zhaohui Li, Yuanqiang Sun, Xin Geng, Yalei Hu, Hongmin Meng, Jia Ge, Lingbo Qu

**Affiliations:** 1 College of Chemistry and Molecular Engineering, Zhengzhou University, Zhengzhou, 450001 China; 2 Institute of Chemical Biology and Nanomedicine, Hunan University, Changsha, 410082 China

## Abstract

Carbon dots (CDs) have a wide range of applications in chemical, physical and biomedical research fields. We are particularly interested in the use of CDs as fluorescence nanomaterials for targeted tumor cell imaging. One of the important aspects of success is to enhance the fluorescence quantum yields (QY) of CDs as well as increase their targetability to tumor cells. However, most of the reported CDs are limited by relative low QY. In the current study, for the first time, one-step synthesis of highly luminescent CDs by using folic acid (FA) as single precursor was obtained in natural water through hydrothermal method. The as-prepared CDs exhibited QY as high as 94.5% in water, which is even higher than most of organic fluorescent dyes. The obtained CDs showed excellent photoluminescent activity, high photostability and favorable biocompatibility. The FA residuals in CDs led to extraordinary targetability to cancer cells and promoted folate receptor-mediated cellular uptake successfully, which holds a great potential in biological and bioimaging studies.

## Introduction

Photoluminescence (PL) nanomaterials, such as carbon dots (CDs), semiconductor quantum dots, upconversion nanoparticles and luminescent metal complexes offering unique chemical and physical characteristics^1–6^, have dramatically changed the paradigm of fluorescence in the past decades. Among them, CDs have emerged as a novel type of PL nanomaterial^7^, which attracted significant and growing interest in both scientific and technology circles due to their excellent chemical and photo-physical properties. CDs have been widely applied in chemo/biosensors, bioimaging, drug delivery, catalysis, optoelectronic devices and white light-emitting diodes, etc.^8–11^ As is well known, CDs with fine performance, especially high fluorescence quantum yield (QY), could maximize their desired applications in those areas. However, efficient methods for the synthesis of CDs with high QY are still remaining as a challenge^12–14^. Most reported CDs are limited by relative low QY. Therefore, extensive efforts have been devoted in the development of high fluorescent CDs. Up to now, chemical doping with heteroatoms is the major renowned pathway to tune the intrinsic properties of CDs. Heteroatoms such as P, B, N and S have been studied to be doped in CDs^15–18^. For instance, nitrogen-doped CDs (N-CDs) showing a QY of 80% was synthesized using citric acid and ethylenediamine by a hydrothermal method^10^. Most recently, Nitrogen and phosphorus co-doped CDs (N,P-CDs) with QY of 17.5% were prepared by microwave-assisted thermolysis of N-phosphonomethyl aminodiacetic acid and ethylenediamine^12^. Peng’s group prepared N-CDs under nitrogen atmosphere by using a sand bath treatment of citric acid in the presence of 4,7,10-trioxa-1,13-tridecanediamine (TTDDA), and the obtained N-CDs exhibited stronger PL with QY up to 33.9%^13^. Cysteine was used to produce sulfur doped CDs (S-CDs) with QY of 73% using hydrothermal method^14^. Among them, N-doped CDs, which can be inherited from nitrogen rich precursors such as low cost and environment-friendly amino acids, proteins and other nitrogen-containing natural products^19,20^, are more attractive due to their excellent optical properties especially increased fluorescence QY.

**Figure 1:**
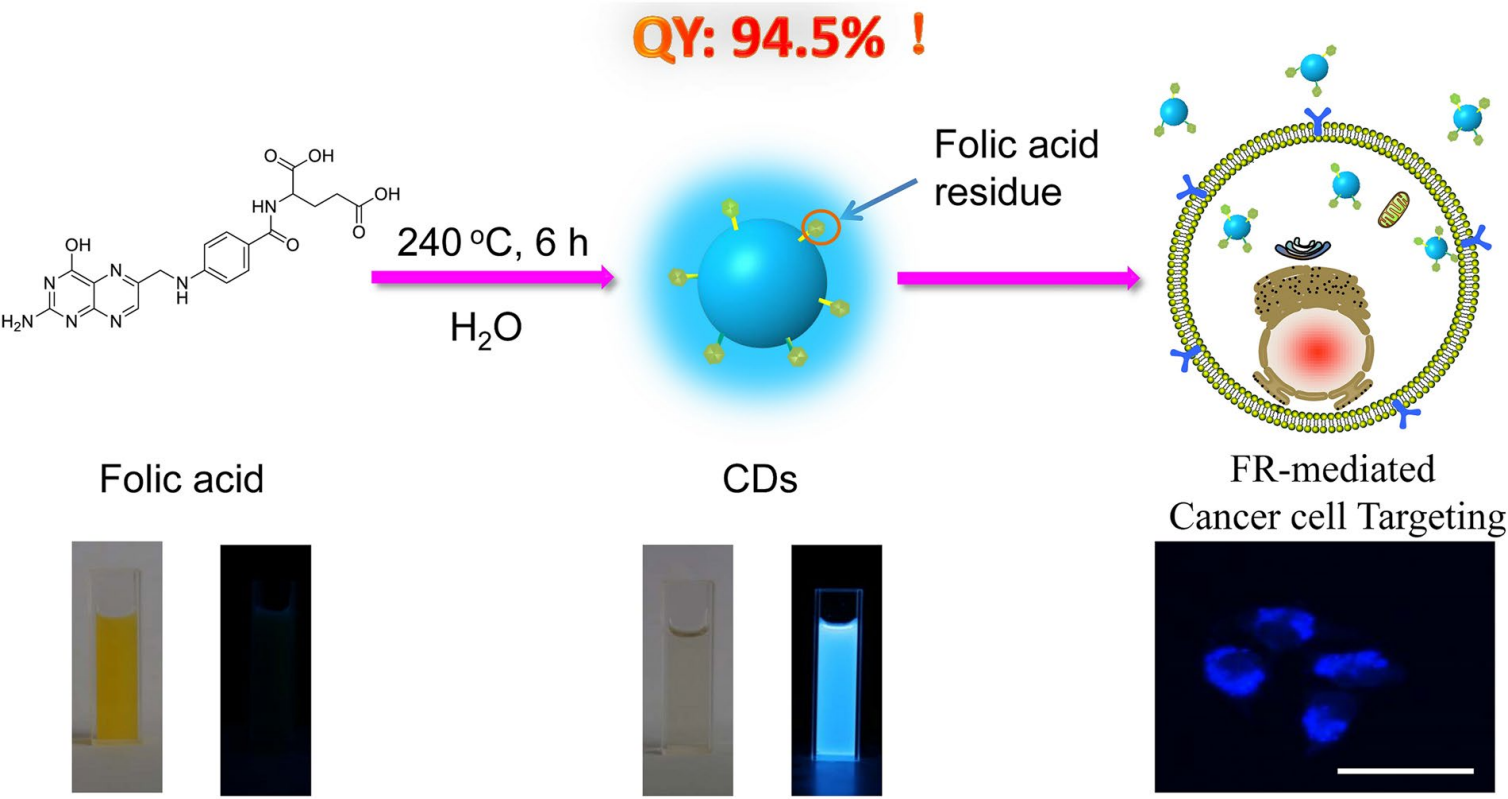
Schematic illustration of the synthesis of folic acid (FA)-derived CDs and their application for folate receptor (FR)-mediated cancer cell targeting.

Folic acid (FA), one of the B vitamins, is an essential dietary component that can effectively decrease the risk of several diseases^21,22^, such as neural-tube defects and congenital heart defects. Particularly, FA has a good targetability to cancer cells^23,24^. Consisting rich nitrogen and functional groups of –OH, –NH_2_ and –COOH, FA has a great possibility to be sufficiently condensed, polymerized and carbonized to generate N-doped CDs without addition of any additives or passivation agents. Herein in this study, FA was chosen as a single precursor to prepare N-doped CDs by hydrothermal-assisted method. Briefly, FA was directly dissolved in ultrapure water without addition of any additives and simply followed by a hydrothermal treatment at melting point 240 °C for 6 h (see Fig. 1 and the experiment section), and ultra-bright FA-derived N-CDs with QY up to 94.5% in water were obtained successfully. Moreover, these CDs showed instinctive targetability to cancer cells without the requirement of any additional surface modifications.

## Results and Discussion

### Structural and morphological properties of N-CDs

**Figure 2:**
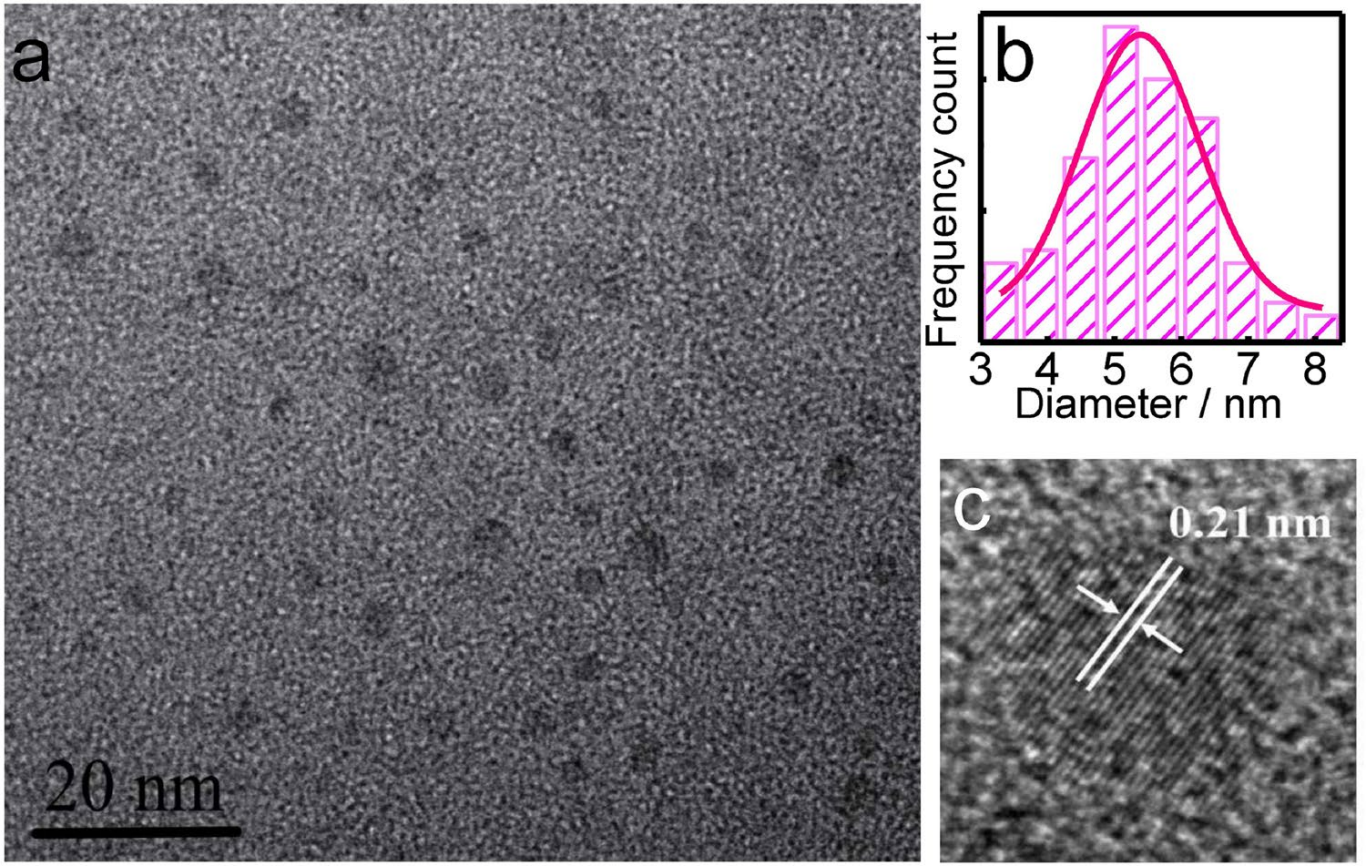
(**a**) TEM graph of the obtained CDs. (**b**) Size distribution of CDs. (**c**) HRTEM image of the CDs showing their lattice spacing.

The morphology of the as-prepared N-doped CDs were characterized. As can be seen from the TEM image in Fig. 2a, the CDs are mostly monodispersed and the particle diameters are between 4 nm and 8 nm with an average size of 5.4 ± 2.2 nm (Fig. 2b). From high-resolution TEM result (HRTEM, Fig. 2c), the image clearly reveals the presence of both crystalline graphite and amorphous phases, and the lattice spacing of 0.21 nm agrees with that of in-plane lattice spacing of graphene (100 facet)^14,25^. As shown in Figure S1, the XRD pattern reveals that one diffraction peak at 2θ of 22.69° corresponds to disordered carbon atoms and the (002) graphite lattice^26^.

### Photoluminescence Peroperties of CDs

**Figure 3:**
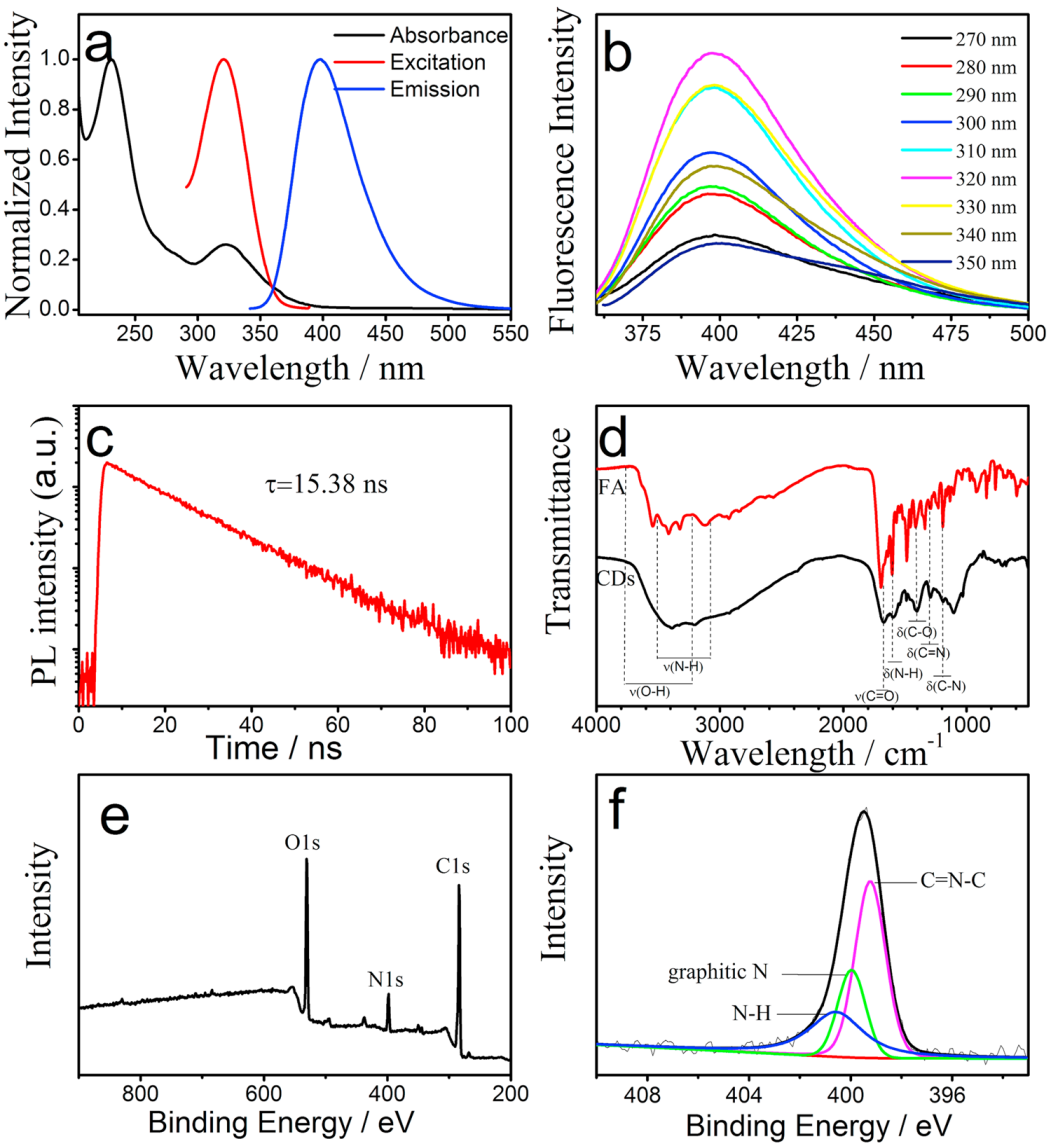
(**a**) UV-vis absorption (black line), PL excitation (red line) and emission (blue line) spectra of the obtained CDs. (**b**) Emission spectra of CDs at different excitation wavelength (start from 270 to 350 nm with a 10 nm increment). (**c**) PL decay curve of CDs. (**d**) FTIR spectra of the precursor (FA, red line) and the prepared CDs (black line). (**e**) XPS spectrum of the prepared CDs. (**f**) High-resolution N1s peaks of the prepared CDs Fig. 1. Schematic illustration of the synthesis of folic acid (FA)-derived CDs and their application for folate receptor (FR)-mediated cancer cell targeting.

Figure 3a shows that the maximum excitation wavelength of the remarkable optical CDs is located at 320 nm, which is consistent with the UV–vis absorption spectrum characterized at 320 nm, giving an emission band characterized at 400 nm. Unlike most other reported CDs, Fig. 3b shows that the emission wavelength of the as-prepared CDs is independent of the excitation wavelength. Excitation-independent FL behavior reflects that the surface state of these CDs are mainly single energy level, and almost all excited electrons return to the ground state via a radiative route^27,28^, which can enhance the passivation degree of traps on CDs and consequently increase the fluorescence QY^29–31^. PL decay curves of the CDs only exhibit a single-exponential function and the fluorescence decay average is 15.38 ns as shown in Fig. 3c. Single lifetime of as-prepared CDs reveals the high ratio of radiative-to-nonradiative transition, which benefit the generation of high QY CDs. The calculations of radiative transition rate constant (Kr) and nonradiative transition rate constant (Knr) were shown in Table S1. Apparently, the Kr (6.14 × 10^7^ S^−1^) is much larger than Knr (0.36 × 10^7^ S^−1^), and consequently high QY was obtained.

Furthermore, the surface composition and chemical state of the obtained CDs were investigated via FTIR and XPS analysis, respectively. As shown in Fig. 3d, the asymmetric stretching vibrations at 1250 cm^−1^ and bending vibrations at 1315 cm^−1^ are attributed to C–N and C=N of the pterin ring. The absorption peak at 3421 cm^−1^ corresponds to O–H stretching vibration. The absorption peak at 3387 cm^−1^ can be assigned to the N–H stretching vibration, whereas the absorption peaks at 1670 cm^−1^ and 1402 cm^−1^ are attributed to C=O stretching vibrations and C–O bending vibration, respectively. All the chemical bonds in the as-prepared CDs were also clearly observed in their HRXPS spectrum. Figure 3e indicates that the as-prepared CDs are mainly composed of three elements of C, O and N. In the high-resolution N1s spectrum (Figure 3f), the three fitted peaks at 399.1, 399.6, and 400.6 eV can be assigned to C=N–C, graphitic N, and surface NH_2_ groups of CDs, respectively. During the investigation via FTIR and XPS analysis, C–N, C=N and C=N–C was found on the partical surface, which indicated the existing of folic acid redidues realizing the cancer cell targebility of the as-prepared carbon dots^32,33^. O1s and N1s peaks suggest that CDs are wrapped outside containing –NH_2_, –COOH, –OH and pterin groups. –NH_2_ and –COOH of FA molecules have high reaction efficiency under hydrothermal condition. The resulting CDs combined with the advantages of N-doping exhibit strong PL emission. The rich amino groups on the CDs surface might contribute to the ultra-high QY. The amino-groups on the surface of CDs can enhance the conjugation degree of conjugated systems, which increase the electron transition probability from the ground state to the lowest excited singlet state, and finally contribute to higher QY of the CDs indirectly^14^.

Figure S2a shows that the resulting CDs possess robust stability and preserve stable fluorescence at pH between 4 and 11 in Britton–Robinson buffer. Moreover, compared with the reported fluorescent materials, the as-prepared CDs showed superior photostability. After 150 min of continuous irradiation by a 150 W xenon lamp, approximate 92% of the initial intensity is still retained (Figure S2b). In addition, the obtained CDs solution exhibits a long-term homogeneous phase without any noticeable precipitation at room temperature, and maintains strong as well as stable fluorescence for more than 5 weeks while being stored in ambient environment (Figure S2c). Furthermore, to investigate whether the as-prepared CDs is stable in complicated circumstance, the effects of common cations (Na^+^, Zn^2+^, Ca^2+^, Mg^2+^, Fe^3+^, Fe^2+^), sugars (maltose, sucrose, fructose, galactose), IgG, BSA, ATP, GSH, and amino acids on the fluorescence response of the CDs were recorded by fluorescence spectrophotometer. As shown in Figure S3, there is no obvious change in the fluorescence intensity for CDs. These results indicate that these CDs have very good photo- and chemostability. Moreover, owing to the favorable biocompatibility of folic acid, the as-prepared CDs exhibit little toxicity to human Hela cells (Figure S4) and A549 cells (Figure S5).

### Targeted cancer cell imaging by CDs

**Figure 4:**
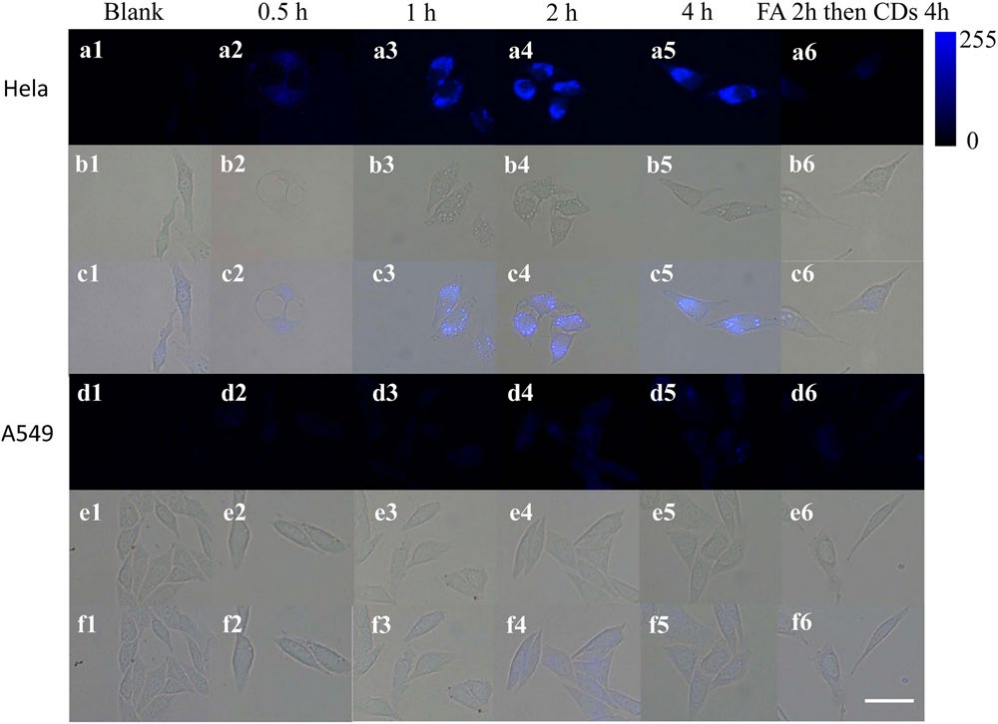
Fluorescence images of Hela and A549 incubated with the FA derived CDs (100 μg/mL) at 37 °C for different incubated time, 0 h (**a1,d1**), 0.5 h (**a2,d2**), 1 h (**a3**,**d3**), 2 h (**a4,d4**), and 4 h (**a5,d5**). As another control, Hela and A549 pre-treated at 37 °C for 2 h with excess FA for FR saturation, and then incubated with CDs (100 μg/mL) at 37 °C for 4 h (**a6,d6**). Bright field images are shown in b and e. The overlay images are shown in c and f. Scale bar: 10 μm.

Due to the ultra-high QY, robust stability, favorable biocompatibility and FA-induced targetability, the as-prepared CDs has a great possibility to be utilized for biological and biomedical applications. As a proof of concept, the aqueous solution of CDs was used for cancer cell imaging in this study. Hela cells, overexpressing folate receptor (FR), were selected as a model. As indicated in Fig. 4a, fluorescence microscopy images demonstrate that CDs can readily enter into Hela cells. When the cells were incubated with CDs for only 1 h, bright fluorescence was observed (Figure 4a3). Further increment of incubation time, only a slight brightness enhancing was shown. Because of the overexpressing of FR, Hela cells can bind and uptake the CDs with FA residues on the surface in a short time. As shown in Fig. 4c, A549 cells, which are deficient in FR expression on their surface^34^ and selected as control cells, are scarcely fluorescence labeled following incubation with the CDs for 1 h. When being incubated with CDs for 4 h, A549 cells still show much lower fluorescence intensity than Hela cells, and the corresponding statistical analysis (Figure S5) clearly shows that folate residues constitute the essential factor for cell targeting and uptaking of the CDs. As another control, a relatively weak fluorescence image was obtained when Hela cells were pretreated with excess free FA and subsequently incubated with CDs (100 μg/mL) for 4 h (Fig. 4a6). Correspondingly, A549 cells blocked with FA exhibits no essential change in fluorescence image (Fig. 4c6). Furthermore, when folate receptor positive SKOV 3 cells are incubated with CDs, similar results with Hela cells can be obtained (Figure S4). These results demonstrated that FA residues in CDs constituted the essential factor for cellular targeting and uptaking, showing that the FA-derived N-CDs can effectively target FR-positive cancer cells via a FR-mediated process.

### Possible Origins for Photoluminescence Superiority of CDs

**Figure 5:**
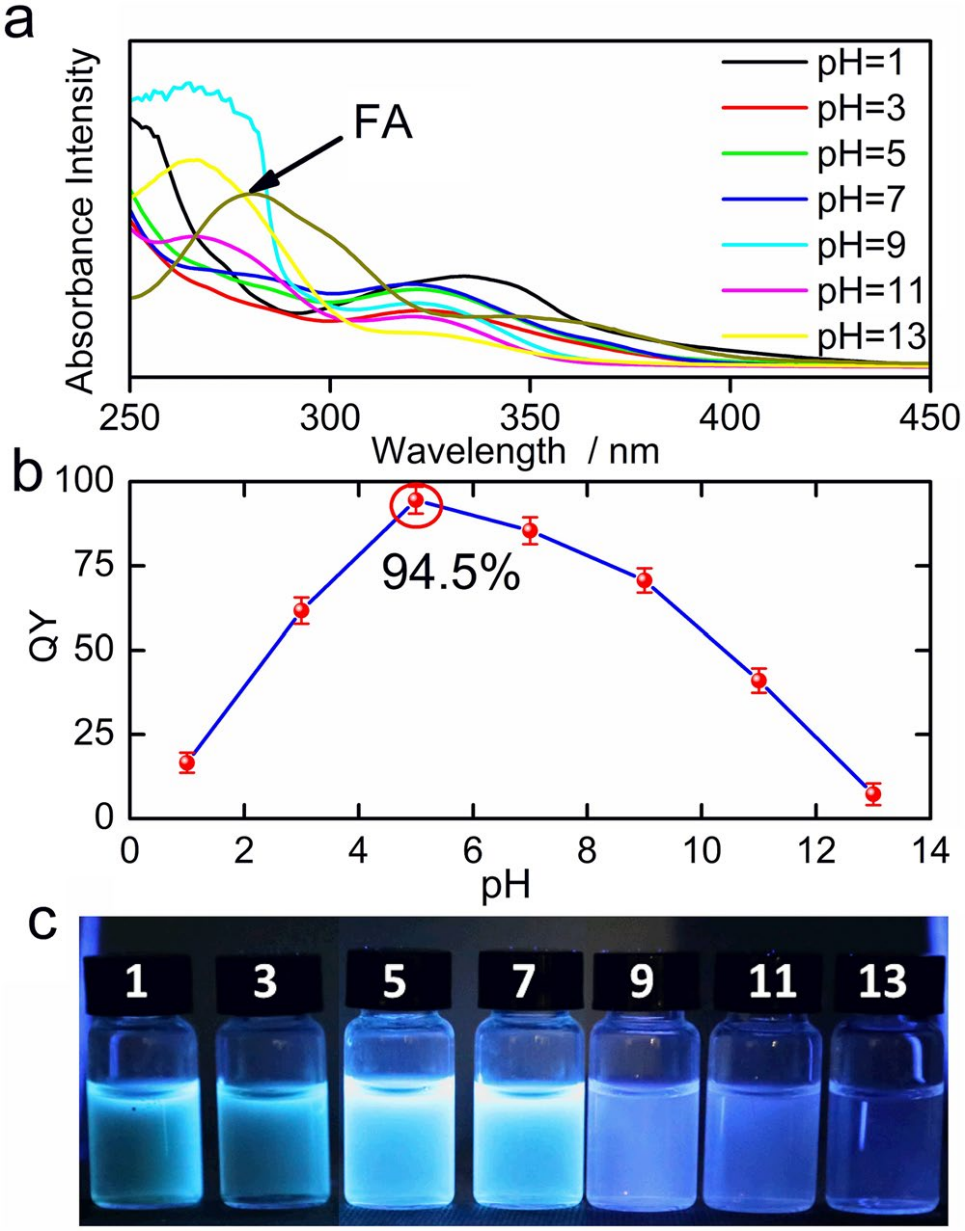
(**a**) Absorption spectra of the as-prepared CDs from FA under different pH. (**b**) Quantum yields of diverse carbon dots and (**c**) the corresponding photos.

To further explore the outstanding optical property of ultra-high QY, the influence of pyrolysis temperature was investigated firstly. When the pyrolysis temperature was near the melting point of precursor FA (240 °C), the highest fluorescence QY was obtained (Figure S6), which is in agreement with the report that CDs with high QY would like to format when heating the small organic molecules above their melting point^9^. Meanwhile, Fig. 5 shows that the pH value of the reaction solution is vital important for the formation of ultrahigh QY CDs. As shown in Fig. 5a, the UV-vis absorption peak around 280 nm of as-prepared CDs is higher in the strong basic or acid condition, where the absorption peak around 280 nm is consistent with the π–π* transitions of the aromatic sp^2^ carbons within the CDs’ core and the absorption of the precursor FA (Figure S9), which cannot produce observed fluorescence signal. In other words, the strong absorption around 280 nm means that the hydrothermal reaction was incomplete in strong basic or acid condition. Although the strong basic or acid condition could improve the solubility of FA at room temperature, the FA might be breakdown and the partial activity of FA’s residues were shielded, resulting in the synthesized CDs with relative low QY. In our current work, with the reaction temperature is near the melting point of FA, the hydrothermal reaction in a closed system has higher efficiency that FA-derived CDs would like to possess an ultrahigh QY without further treatment to assist the solubility of FA. Obviously, the weak acid circumstance of 5.0, which was obtained directly by dissolving FA in nature water, is the key factor for the synthesis of ultra-bright CDs (Fig. 5b and c). When the experiment was carried out in natural water, partial activity of –OH was shielded^35^, and the dehydration condensation was mainly between the carboxyl and amino groups of FA. Compared with partial shielding of –NH_2_ and –COOH groups in strong basic or acid conditions, the condensation of FA in natural water is much more effective and consequently the QYs of CDs is much higher. Therefore, circumstance condition of pH is critical valuable for CDs synthesis and directly impact on the fluorescence intensity of the obtained CDs.

## Conclusion

To summarize, ultrahigh fluorescent FA-derived CDs were synthesized through a facile one-step and green preparation of hydrothermal approach by importing FA as carbon source, nitrogen source and surface passivation agent. The as-prepared CDs show excellent PL activity and excitation-independent emission. These nanoparticles offer several distinct advantages: (1) the high fluorescence QY reaches up to 94.5%; (2) these CDs have robust photostability, chemostability, and biocompatibility; (3) the residues of FA on CDs can specifically recognize and target FR, and malignant cancer cells with FR expressing can be easily distinguished. Overall, these ultra-bright CDs has a great potential to be widely useful in a number of biological studies and biomedical applications.

### Experimental Procedures

#### Apparatus and Characterization

UV-vis absorption spectra were recorded on a TU-1810 spectrophotometer (Beijing, China) in 1 cm quartz cells. Fluorescence measurements were recorded by using an F-4600 spectrophotometer (Hitachi, Japan). Fluorescence QY was measured directly by using the absolute QY measurement system on a FLS 980 fluorometer (Edinburgh Instrument, British) equipped with an integrating sphere (IS). The IS consists of a 120 mm spherical cavity. Fourier transform infrared (FTIR) spectrum was characterized at room temperature by a Bruker Tensor 27 spectrophotometer (Bruker, Germany). Morphological details and sizes of CDs were determined by using a transmission electron microscope (FEI-Tecnai G2, USA). The crystallinity was determined using a powder X-ray diffractometer (PANalytical, Netherlands). The X-ray photoelectron spectroscopy (XPS) analysis was performed by using an ESCALAB 250 photoelectron spectrometer (Thermo Fisher Scientific, USA) with Al Kα (1486.6 eV) as the X-ray source. Cells were imaged using an inverted fluorescence microscope (Nikon, Japan) equipped with a 100 W mercury lamp (C-SHG1, Nikon, Japan).

#### Preparation of N-doped CDs

The N-doped CDs were obtained by a hydrothermal method using FA as single precursor. The experimental procedure is described as follows. FA (0.0050 g) was dissolved in 7.5 mL ultrapure water. The solution was sonicated for 5 min to form a well-distributed solution and then heated in an oven for 6 h at 240 °C for carbonization. After this reaction, a clear light yellow-brown solution was obtained. The solution was centrifuged for 10 min at 10,000 rpm to remove precipitate. The obtained CDs were purified by column chromatography on a Sephadex G-25 gel filtration column (1 × 20 cm) eluted with water for separation. Size exclusion chromatogram of as-synthesized CDs solution was shown in Figure S12, all the fluorescence CDs product eluted between 20 and 80 min was collected as purified CDs followed by being lyophilized and stored at room temperature for further use. To investigate the influence of pH value of the FA solution, series FA CDs share the same synthetic route but with the addition of HCl or NaOH for adjusting the pH value of the FA solution before hydrothermal synthesis.

#### Cytotoxicity assay

The cytotoxicity of CDs was evaluated on HeLa and A549 cells using a standard MTT assay. Briefly, HeLa and A549 cells were seeded in 96-well plates at a density of 5 × 10^3^ cells per well in a culture medium and allowed to grow over 24 h (the cells reached 70–80% confluence). After the medium was replaced with fresh RPMI 1640 (90 μL), different amount of CDs were added to keep the final concentration in the range of 0–350 μg/mL for incubation at 37 °C for 24 h. Then, the culture media were discarded. MTT solution (20 μL, 0.5 mg/mL^−1^ in RPMI) was added to each well. The cells were incubated for another 4 h and then the supernatant was abandoned. DMSO (150 μL) was used to liberate the formed formazan. After shaking the plate for 10 min, the absorbance at 490 nm was measured to calculate the cell viability rate (VR) according to Equation (1):

1 $$VR=A/{A}_{0}\times 100{ \% }$$

(where A is the absorbance of the experimental group (the cells were treated with CDs) and A_0_ is the absorbance of the control group).

The cells treated with PBS and DMSO, which did not contain CDs, were taken as the control group. Five parallel samples were tested in each group and three independent experiments were performed under identical conditions.

#### Targeted tumor cell imaging

Tumor cells were seeded in culture dishes and cultured at 37 °C using RPMI 1640 growth medium with 10% fetal bovine serum, 100 units per mL penicillin, and 100 μg/mL streptomycin. Cells were then incubated in a humidified incubator at 37 °C with 5% CO_2_. All the cells were incubated at approximately 70% confluence until normal morphology was achieved. Then, the mixture of CDs (100 μg/mL) in RPMI 1640 medium was added to each dish. The cell dish was placed in the incubator at a desired time. The cells were washed thrice with 0.1 M phosphate-buffered saline (PBS) solution (pH = 7.4) to remove unassociated compounds. Finally, the cells were used for fluorescence imaging. Competition experiments were conducted in the cell culture pre-treated with 50 μL of saturated FA solution for 2 h prior to CDs treatment to confirm the receptor-mediated uptake.

## Electronic supplementary material

Supplementary Information
