# Supplementary material for: Synthesis of Luminescent Carbon Dots with Ultrahigh Quantum Yield and Inherent Folate Receptor-Positive Cancer Cell Targetability
Source: Sci Rep. 2018 Jan 18;8:1086. doi: 10.1038/s41598-018-19373-3 (PMC5773692; doi:10.1038/s41598-018-19373-3)
Supplement: Supplementary file 1 — Supplementary Information [file 41598_2018_19373_MOESM1_ESM.pdf]

Supporting Information

Synthesis of Luminescent Carbon Dots with Ultrahigh Quantum Yield and Inherent Folate Receptor-Positive Cancer Cell Targetability

Haifang Liu[1], Zhaohui Li[1,2,*], Yuanqiang Sun[1], Xin Geng[1], Yalei Hu[1], Hongmin Meng[1], Jia Ge[1], and Lingbo Qu[1,*]

[1] College of Chemistry and Molecular Engineering, Zhengzhou University, Zhengzhou 450001, China

[2] Institute of Chemical Biology and Nanomedicine, Hunan University, Changsha 410082, China

*Corresponding authors: zhaohui.li@zzu.edu.cn (Z. H. Li), qulingbo@zzu.edu.cn (L. B. Qu).

Table of Contents

1. **Figure S1.** XRD pattern of the obtained CDs.
2. **Figure S2.** Stability investigation of CDs.
3. **Figure S3.** The stability of CDs.
4. **Figure S4.** Fluorescence images of SKOV 3 incubated with the FA derived CDs.
5. **Figure S5.** Emission intensity plot calculated through summation of the fluorescence intensity associated with the Hela, SKOV 3 and A549 cells.
6. **Figure S6.** Effects of CDs with varied concentrations on the viability of Hela cells.
7. **Figure S7.** Effects of CDs with varied concentrations on the viability of A549 cells.
8. **Figure S8.** Absorption spectra and quantum yields of the CDs derived from FA under different temperature.
9. **Figure S9.** Absorption spectra and quantum yields of the CDs derived from FA under different reaction time.
10. **Figure S10.** Absorption spectra and quantum yields of the CDs derived from FA under different concentrations of FA.
11. **Figure S11.** Absorption spectra of the as-prepared CDs and folic acid.
12. **Figure S12.** Size exclusion chromatogram of as-synthesized CDs solution.
13. **Table S1.** The conventional relationships analysis of Kr and Knr.

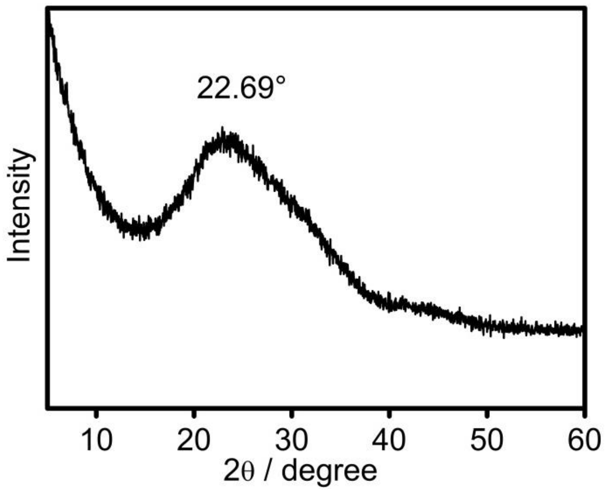

Figure S1. XRD pattern of the obtained CDs.

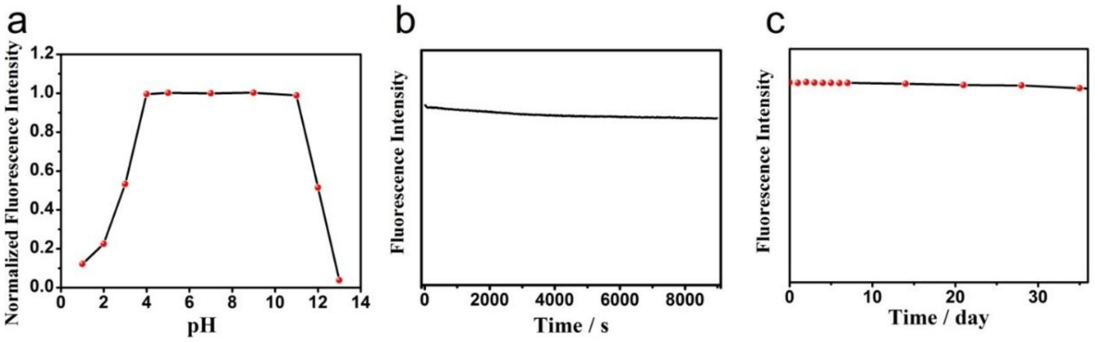

Figure S2. Stability investigation of CDs. a) Effect of pH on the fluorescence intensity of CDs, the detecting solutions were prepared in Britton–Robinson buffer and the pH was adjusted by using HCl or NaOH. b) Photostability of CDs aqueous solution under continuous irradiation by a 150 W xenon lamp. c) Temporal evolution of fluorescence of the CDs during storage for 35 days.

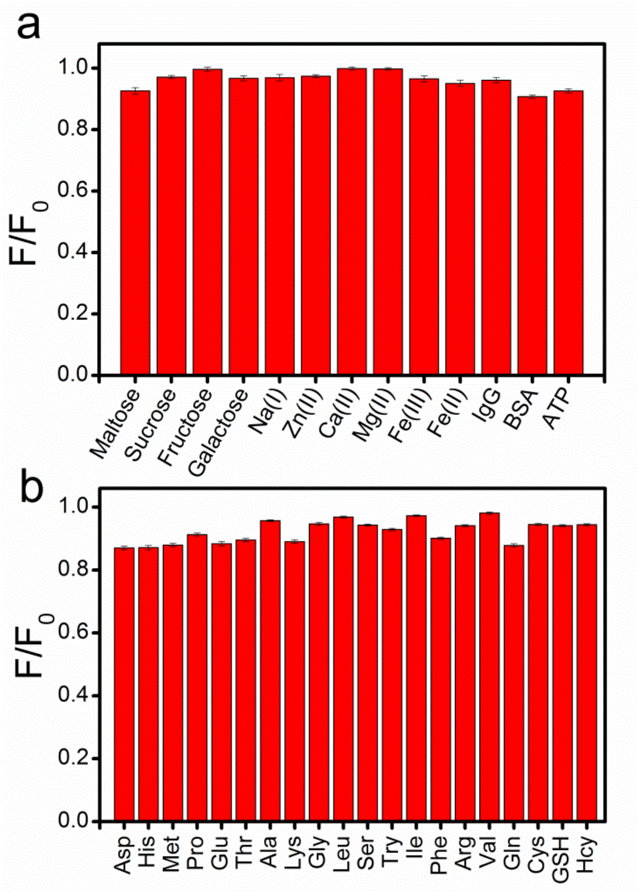

Figure S3. The circumstance stability of CDs. a) The effects of common cations (Na^{+}, Zn^{2+}, Ca^{2+}, Mg^{2+}, Fe^{3+}, Fe^{2+}), sugars (maltose, sucrose, fructose, galactose), IgG, BSA, ATP, GSH, and b) amino acids on the fluorescence response of as-prepared CDs.

Data notes: To investigate whether the prepared CDs are stable for some common ions and molecules, the effect of 4 kinds of sugar with the concentration of 0.1mM, 6 kinds of cations with the concentration of 0.01 M, 2 kinds of proteins (IgG 2mg/mL, BSA 0.1mg/mL), ATP(1 μM), GSH(1 mg/mL), and 19 kinds of amino acids with the concentration of 0.1 mg/mL on the fluorescence response of as-prepared CDs were recorded by fluorescence spectrophotometer.

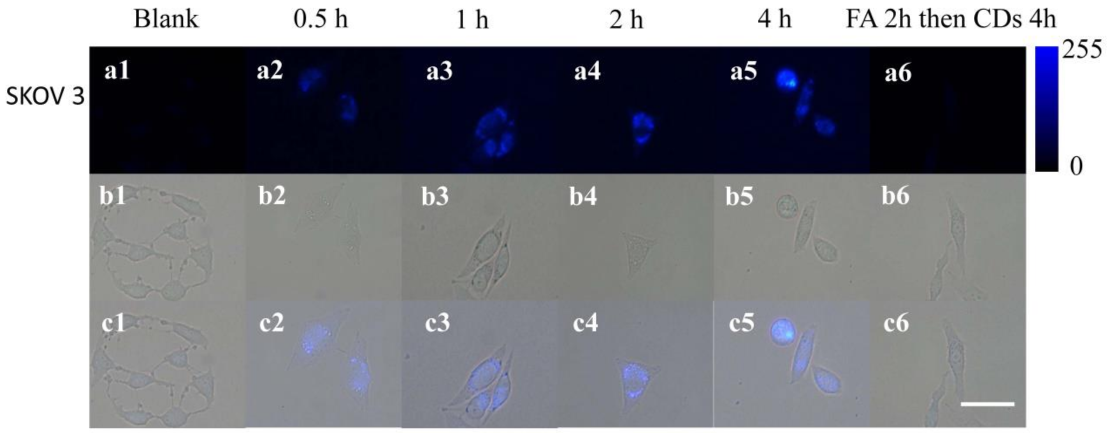

Figure S4. Fluorescence images of SKOV 3 incubated with the FA derived CDs (100 μg/mL) at 37 °C for different incubated time, 0 h (a1), 0.5 h (a2), 1 h (a3), 2 h (a4), and 4 h (a5). As a control, SKOV 3 cells pre-treated with excess FA at 37 °C for 2 h for FR saturation, and then incubated with CDs (100 μg/mL) at 37 °C for 4 h (a6). The bright field images are shown in the second line (b), and the overlay images are shown in the third llin (c). Scale bar, 10 μm.

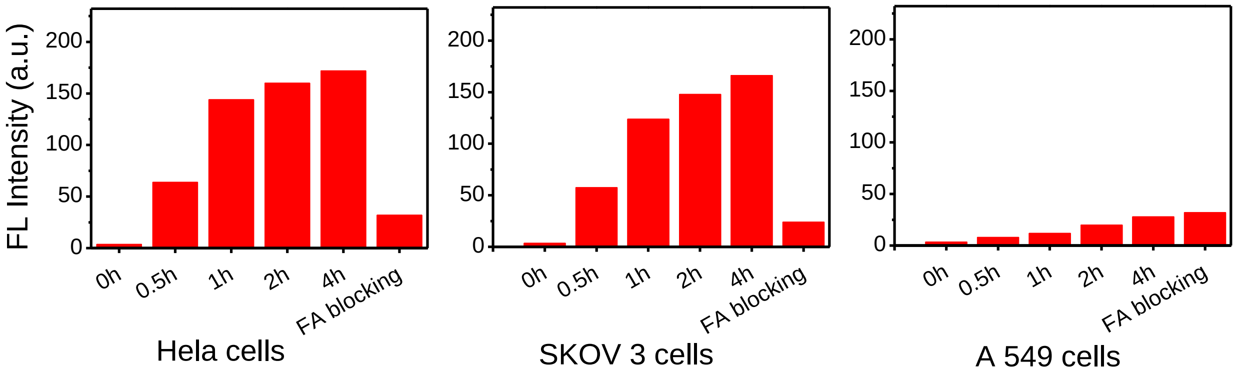

Figure S5. Emission intensity plot calculated through summation of the fluorescence intensity associated with the Hela (left), SKOV 3 (middle), and A549 cells (right). The overall fluorescence intensity was normalized by the number of cells and cell area.

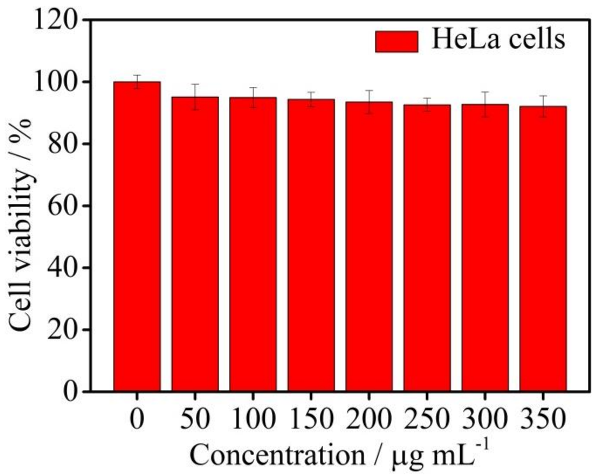

Figure S6. Effect of CDs with varied concentrations on the viability of Hela cells (the viability of the cells without CDs is defined as 100%). The results are the mean SD of five separate measurements.

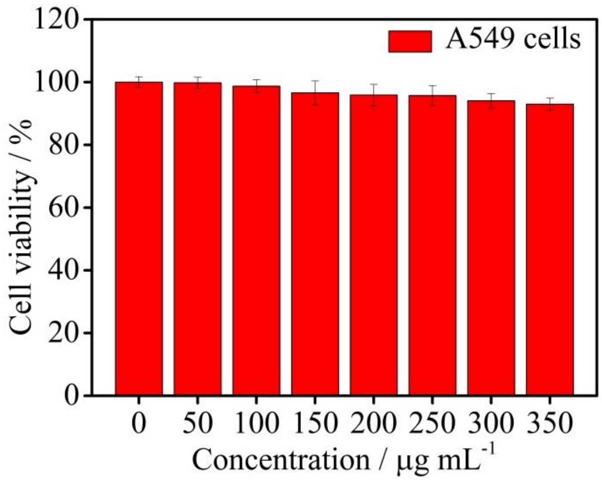

Figure S7. Effect of C-dots with varied concentrations on the viability of A549 cells (the viability of the cells without C-dots is defined as 100%). The results are the mean SD of five separate measurements.

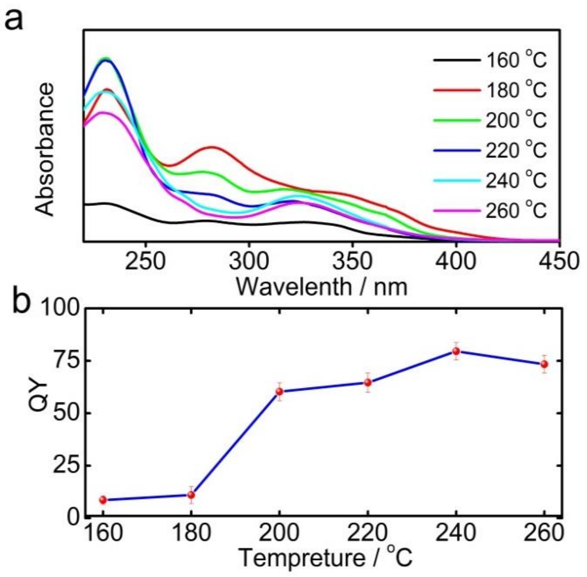

Figure S8. a) Absorption spectra and b) quantum yields of the CDs derived from FA under different temperature, the reaction time is 10 h and the concentration of FA is 3 mg/mL.

Data notes: The influence of pyrolysis temperature was investigated,[1] and the result shows that the pyrolysis temperature is vital important for the formation of CDs. As shown in Figure S6b, when pyrolysis temperature is lower than 180 °C, the PL QYs of as-prepared CDs is less than 9%. However, when the pyrolysis temperature is higher than 200 °C, the PL QY of most prepared CDs increases sharply from 11% to 80% with the increment of temperature. When the pyrolysis temperature is near the melting point of FA (240 °C), the highest fluorescence QY of 79.6% can be obtained. Further increment of temperature form 240 to 260 °C results in a slight decrement of QY. Therefore, 240 °C was used for the following experiments. Meanwhile, UV-vis spectra of the CDs were further investigated. As can be seen from Figure S6a, with the reaction temperature increases, the absorption peak around 280 nm is decreased and the peak around 320 nm becomes sharper. The peak around 280 nm is consistent with the π–π* transitions of the aromatic sp^2 carbons within the CDs' core and the absorption of the precursor FA, which cannot produce observed fluorescence signal. Correspondingly, the absorption peak around 320 nm is from the trapping of excited state energy of the

surface states, which can lead to strong fluorescence. These results demonstrated that more fine-tuned CDs were synthesized with the reaction temperature increasing, which is in agreement with the enhancement of QYs.

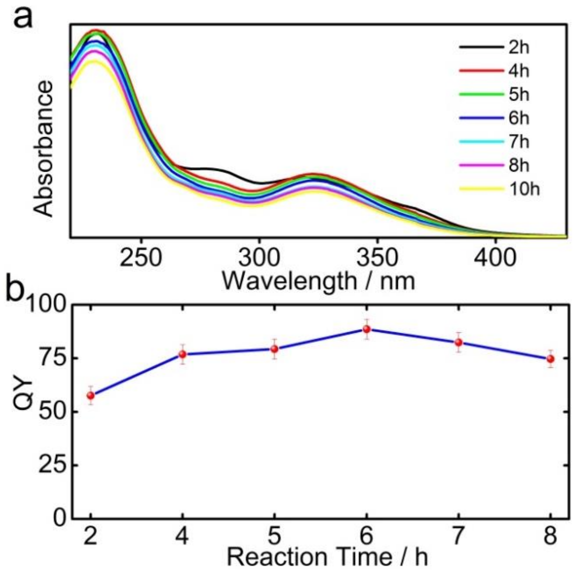

Figure S9. a) Absorption spectra and b) quantum yields of the CDs derived from FA under different reaction time. The concentration of FA is 3mg/mL and the temperature of 240 °C is used for hydrothermal treatment.

Data notes: The reaction time is another important factor for CDs carbonization. As the reaction time increased from 2 h to 6 h at the temperature of 240 °C, the QY increased from 58% to 89% (Figure S7b), which was also in accordance with the UV-vis and PL spectra (Figure S7a). At higher temperature, partial carbogenic CDs were formed because of carbonization. With the reaction time extended, the resulted CDs would be further carbonized and generate stronger fluorescence, which is due to the synergistic effect of carbogenic core as well as surface state, and the carbogenic core plays an ever greater role in CDs as synthesis time increases. Owing to further carbonization, the QY decreased from 89% to 75% as the reaction time continuously increased from 6 h to 10 h. Thus, 6 h was chosen as the optimized reaction time.

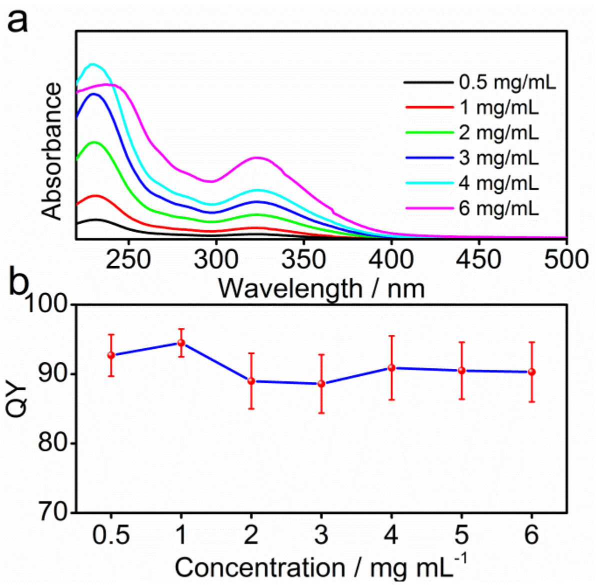

Figure S10. a) Absorption spectra and b) quantum yields of the CDs derived from different concentrations of FA.

Data notes: The concentration of FA is another important factor for CDs carbonization. As the concentration of FA increased from 0.5 mg/mL to 1 mg/mL at the temperature of 240 °C for 6 h, the QY increased from 92.7% to 94.5% (Figure S7b), which is also in accordance with the UV-vis and PL spectra (Figure S8a). The concentration of FA continuously increased from 1 mg/mL to 6 mg/mL, the QY decreased from 94.5% to 90.3%.Thus, 1 mg/mL was chosen as the optimized concentration. Through the above optimization of pyrolysis temperature, reaction time, and the concentration of FA, the QY of CDs as high as 94.5% was obtained by the hydrothermal treatment of FA (1 mg/mL) at 240 °C for 6 h in pure water. The CDs were further employed for characterization and application.

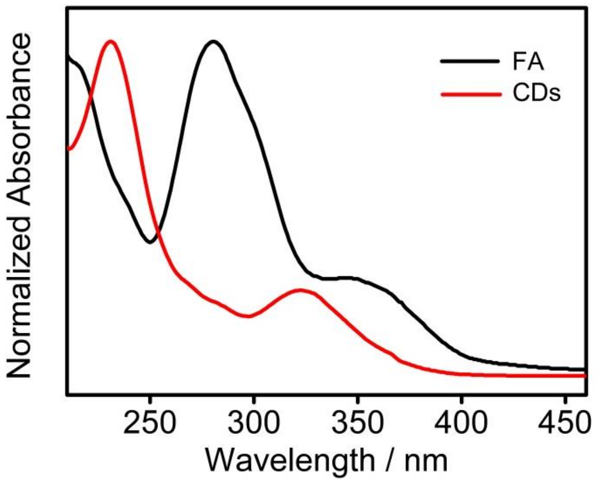

Figure S11. Absorption spectra of the as-prepared CDs (red line) and folic acid (black line).

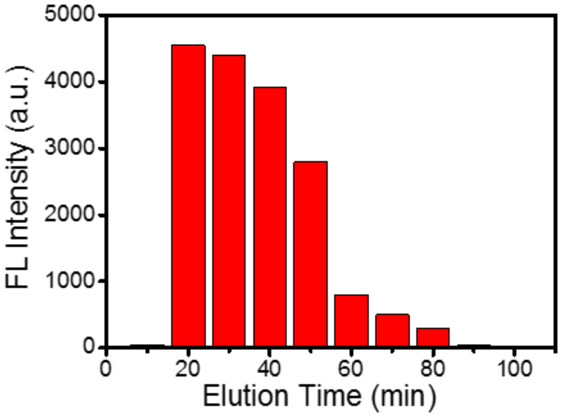

Figure S12. Size exclusion chromatogram of as-synthesized CDs solution. All the fluorescence products were collected as purified CDs from the sub-fractions between the elution time of 20 and 80 min.

Table S1. The conventional relationship analysis of Kr and Knr.

	φ[a]	τ[b]	Kr [c]	Knr [d]
CDs	94.5%	15.38 ns	6.14×10^{7} S^{-1}	0.36×10^{7} S^{-1}

[a] φ: quantum yield.

[b] τ: decay time.

[c] Kr: radiative transition rate constant. Calculation by the equation (1):

Kr =φ / τ [1-2] (1)

[d] Knr: nonradiative transition rate constant, which is the sum of the internal conversion and intersystem crossing rate constants. And calculation by the equation (2):

Knr = (1 –φ) / τ [2-3] (2)

References

[1] D. Stich, F. Späth, H. Kraus, A. Sperlich, V. Dyakonov, T. Hertel, *Nat. Photonics* **2014**, *8*, 139-144.

[2] X. T. Zheng, A. Ananthanarayanan, K. Q. Luo, P. Chen, *Small* **2015**, *11*, 1620-1636.

[3] V. A. Sazhnikov, V. M. Aristarkhov, S. K. Sazonov, A. I. Vedernikov, S. P. Gromov, M. V. Alfimov, *High Energ. Chem.* **2013**, *47*, 339-345.
